# Starch-Based Flexible Coating for Food Packaging Paper with Exceptional Hydrophobicity and Antimicrobial Activity

**DOI:** 10.3390/polym10111260

**Published:** 2018-11-13

**Authors:** Shuzhen Ni, Hui Zhang, Hongqi Dai, Huining Xiao

**Affiliations:** 1 Jiangsu Co-Innovation Center for Efficient Processing and Utilization of Forest Resources, Nanjing Forestry University, Nanjing 210037, China; nishuzhen1988@126.com; 2 Department of Chemical Engineering, University of New Brunswick, Fredericton, NB E3B 5A3, Canada, zhangh10@163.com; 3 College of Materials Engineering, Fujian Agriculture and Forestry University, Fuzhou 350002, China

**Keywords:** starch, ZnO nanoparticles, coating, food packaging paper, hydrophobic, antimicrobial activity

## Abstract

Herein, we fabricated a starch-based flexible coating for food packaging papers with excellent hydrophobicity and antimicrobial properties. FTIR (Fourier transform infrared) and XRD (X-ray diffraction) spectra revealed the homogeneous dispersion of the ZnO nanoparticles (NPs) in the composite film within 5% ZnO NP dosage. SEM (scanning electron microscope) and AFM (atomic force microscope) micrographs confirmed the increased roughness on the composite film with the increased dosages of ZnO NPs. Hydrophobic characteristics showed that dramatic enhancement was obtained in the values and stabilities of DCAs (dynamic contact angles) in the resultant film and coated paper. TG (thermogravimetry) results demonstrated the increased thermal stabilities of the composite films. Significantly, a decreased water vapor transmission rate was observed in the coated paper. When 20% guanidine-based starch and 2% CMC (carboxy methyl cellulose) was added, a flexible coating with excellent antimicrobial activity towards *Escherichia coli* can be obtained. Furthermore, the migration of ZnO NPs into the food simulants was well below the overall migration legislative limit. The resultant starch-based flexible composite film and coated paper established an effective approach to develop a green-based material for food packaging applications.

## 1. Introduction

Increased environmental awareness has impelled various biopolymers to be developed into eco-friendly materials that can replace petroleum-based synthetic materials in the packaging fields [1,2,3,4,5]. As one of the biodegradable polymers, starch has become a promising candidate owing to its abundance, low price and thermoplastic nature [6,7]. However, its application is greatly affected by the intrinsic hydrophilicity and non-resistance to microorganisms.

ZnO nanoparticles (NPs) have been widely applied in the food packaging industry due to the numerous advantages including a broad-spectrum antimicrobial property, UV-shielding capacity, nontoxicity, thermal stability, as well as high specific surface area [8,9]. It has been well developed for constructing a high hydrophobic or superhydrophobic surface via depositing onto various materials in different fields [10]. It is reported that the GaAs surface with an excellent hydrophobicity was obtained via coating with ZnO nanoparticles for its potential application in solar cells, space and optical windows [11]. A reversible super-hydrophobicity to super-hydrophilicity transition on the aligned ZnO films of nanorods was observed under the alternation of UV illumination and dark storage [12]. Superhydrophobic cotton fabrics with a static water contact angle of more than 150° were prepared based on the ZnO nanorod arrays and subsequent hydrophobic modification [13].

Generally, the fabrication of high or super-hydrophobic surfaces focused on the substrates (i.e., metal, paper or cotton) is constructed by totally covering with the ZnO NPs [14,15]. Until now, few studies have been demonstrated for incorporating ZnO NPs in the starch-based coated papers in the food packaging fields. ZnO NPs are believed to be nontoxic, biosafe and biocompatible and have been used in many applications in daily life, such as drug carriers and in cosmetics and fillings in medical materials. They have also been considered as a “GRAS” (Generally Recognized as Safe) substance by the FDA [16]. Based on this, a facile approach has been developed in our studies to improve the hydrophobicity for starch films and papers based on the starch/ZnO/chitosan dispersion system via simple mechanical treatment [17]. However, due to the potential hazards of the nanoparticles for human beings and food safety, the overall migration legislative limits for ZnO NPs should be below 5 mg/kg according to EU No. 10/2011. The rapidly developed toxicity studies of ZnO NPs have shown that the ZnO NPs have cytotoxicity against different culture cells due to the induction of oxidative and inflammatory responses. The cytotoxicity of both bulk and nanoparticles of ZnO in several cell cultures including human lung epithelium, bronchial epithelium, liver, cardiac microvascular endothelial and kidney cells has been studied [18,19,20,21,22]. Heng et al. demonstrated the dose-dependent effects on the cytotoxicity of ZnO NPs on human cell lines [23]. Deng et al. also observed that the toxic effect of ZnO NPs in mouse neural cells was dose dependent rather than size dependent [24]. Therefore, studies on the migration levels of ZnO NPs in food should be conducted.

Antimicrobial packaging plays important roles in delaying food spoilage in food packaging fields. It is a promising form of active packaging and beneficial for both consumers and hygiene industries. Therefore, eco-friendly materials with antimicrobial capacity are in great demand in accordance with the environmental concerns [24,25,26,27,28,29,30]. As such, it is necessary to create a flexible coating for the packaging papers with excellent antimicrobial capacities. Currently, the antimicrobial agents for food packaging include organic compounds (organic acids, alcohols, phenols), inorganic compounds (silver, TiO_2_) and natural compounds (essential oils, lysozyme and bacteriocins). Organic and natural compounds are sensitive to intense processing conditions that are present in the industrial processes (such as high temperatures and pressures) and the development of microorganism resistance [31]. In comparison with the current inorganic antimicrobial compounds, UV irradiation is not needed for the antimicrobial activities of ZnO NPs, and no colour variability problem exists.

The antibacterial activity for the ZnO NPs has been proposed as the release of reactive oxygen species and Zn^2+^ ions, mechanical damage and cellular internalization [32], which can induce the penetration of the cell envelope and disorganization of the bacterial membrane upon contacting ZnO nanoparticles, which can inhibit bacterial growth [33]. Currently, the most reported antibacterial activity of ZnO is ascribed to the separate ZnO particles well dispersed in water, rather than embedded in polymers as a ZnO-polymer composition [34]. Thus, guanidine-based cationic polymers that have shown highly effective antimicrobial, antifungal and antiviral properties with excellent safety to humans are chosen [35,36,37]. The biocidal activity is expressed by its adsorption on the bacterial cell membrane with a negative charge via electrostatic adsorption [38]. Pores are thus formed on the damaged cell membrane and induce the consequent leakage of intracellular component that cause the final lysis of bacterial cells [39,40]. Here, guanidine-based starch is added to our formulations to endow the coated papers with excellent antimicrobial activities.

Therefore, we aim to develop a flexible coating for food packaging papers with significantly improved hydrophobicity and excellent antimicrobial activity. In this work, carboxymethyl cellulose (CMC) was used as the capping agents of ZnO NPs to improve the miscibility between the starch and the ZnO NPs and the flexibility of the starch coating layer due to its excellent flexibility and film-forming ability. Thus, a highly flexible starch coating for food packaging papers was successfully prepared to acquire exceptional hydrophobicity and antimicrobial activity. The surface morphology, miscibility of ZnO and starch, transmittance, thermal stabilities, hydrophobicity and solvent-resistance of the composite film were characterized to reveal the chemical-physical performance of the starch-based coating layer. The antimicrobial activities and migration levels of ZnO NPs into the food simulants for the coated papers were tested. The resulting hydrophobic and antimicrobial packaging paper is very promising for its high-value added applications in the food packaging fields.

## 2. Materials and Methods

### 2.1. Materials and Chemicals

**Figure 1:**
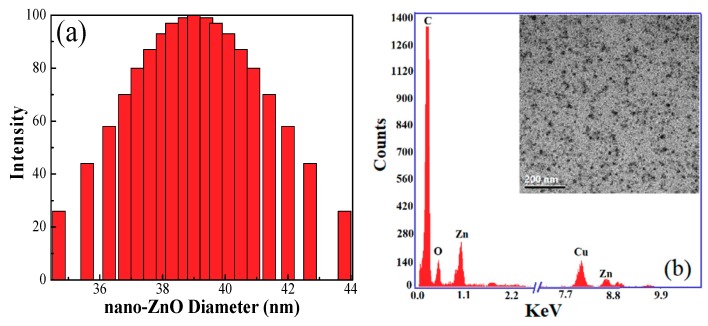
(**a**) The diameter of ZnO NPs; (**b**) the energy dispersion spectrum (EDS) analysis of TEM on the ZnO NPs.

Corn starch was purchased from Ingredion Canada Incorporated (Mississauga, ON, Canada). ZnO nanoparticles with a diameter of 38~40 nm were provided by Sinopharm Chemical Reagent Co., Ltd (Wuxi, Jiangsu, China). Their diameter and TEM morphology were shown in Figure 1. Common filter paper was obtained from Fisher Scientific (Qualitative P8, porosity: coarse, flow rate: fast, Ottawa, ON, Canada). Chitosan with a low molecular weight (*M*_w_ 50,000~190,000, degree of deacetylation of 85.9%), carboxymethyl cellulose (CMC, average *M*_w_ of 250,000), sodium chloride, dimethylacetamide (DMAc), methanol, acetone and other chemicals were purchased from Sigma-Aldrich (Oakville, ON, Canada) and were used without further purification.

### 2.2. Fabrication of Starch-Based Composite Film and Coated Papers

The 2 wt % corn starch solution was prepared by dissolving 20 g starch in 80 g water and heated at 90 °C for 30 min. Two percent (wt) CMC was added and employed as the capping agent to facilitate the adhesion of ZnO NPs onto the surface of starch. After the CMC was uniformly distributed in the starch solution under magnetic stirring, the well-dispersed ZnO NPs (0.4 wt %)) suspension at different addition levels (1%, 3%, 5%, 7% and 9%) were slowly dropped in, followed by the addition of 5 wt % chitosan after the even dispersion of the ZnO NPs. Then, the resultant suspension was magnetically stirred at 300 rpm and subjected to sonication (output at 42 kHz, 100 W, Branson Ultrasonic Corporation, 2510R-DTH, Qsonica, Newton, CT, USA) for 30 min, respectively. The composite films with a base weight of 50 g/m^2^ were fabricated by a facile solution-cast method, and were dried 72 h at room temperature before being peeled off. The common filter paper was coated by the starch suspension on the paper coating machine at the dosage of 4 g/m^2^ followed by 5 min of drying in a quick-drying machine. Guanidine-based starch (i.e., antimicrobial thermal plastic starch, ATPS) with 10, 15 and 20 wt % was added to replace the corresponding corn starch on the original formulations to endow the starch-based coated papers with antimicrobial capability. The specific steps to prepare the ATPS can be seen in our previous study [41]. The water content of the resultant films was around 13 wt % of dry starch.

### 2.3. Characterization

The surface hydrophobicity of the films and papers was measured by the T200 Auto 3 Plus Contact angle analyzer () under ambient conditions. A water drop of 3 μL was deposited on the sample surface, and then, the images were captured and recorded.

Fourier transform infrared spectroscopy (FTIR) was performed on a Nicolet 6700 FTIR spectrometer (Thermo Scientific, Nicolet, QC, Canada) at a resolution of 4 cm^-1^ in the range of 500–4000 cm^−1^, scanning 14 times. The crystallinity of the starch films was recorded by a Bruker D8-Advance X-ray spectrometer (AXS Company, Karlsruhe, Germany) over a 2θ range from 5–80°.

The GENESYS 10 UV–Vis spectrometer (Thermo Fisher Scientific Inc., Waltham, MA, USA) was used to record the light transmittance and absorbance of the films within the UV–Vis region (200–800 nm) using air as the background correction.

The thermal properties of the films were determined on the thermogravimetric analyzer (Q600, TA Instruments, New Castle, DE, USA) in the temperature range from 25–600 °C at a heating rate of 10 °C/min, with a nitrogen flow rate of 10 mL/min continuously passed into the furnace.

The surface morphology of the starch films was characterized by the JEOL 6400 scanning electron microscopy (SEM, JEOL, Tokyo, Japan) at a 15-kV acceleration voltage after gold sputtering. Atomic force microscope (AFM, Santa Barbara, CA, USA) imaging was used to characterize the surface topography and roughness of the films by the MFP-3D AFM (Asylum Research, Santa Barbara, CA, USA) operating in tapping mode under ambient air conditions.

The film’s solvent resistance towards DMAc, methanol, acetone and water was tested by immersing the film samples (10 mm × 50 mm) in the corresponding solvent for 48 h, and photographs were taken after a certain time to show their solvent resistance performance. The solvent absorption of the films was determined by weighing the film strips and calculated by the following equation:

Solvent uptake (%) = [(*w*_2_ − *w*_1_)/*w*_1_] × 100 (1)

where *w*_2_ is the film weight after immersing 48 h in solvent and *w*_1_ is the initial film weight conditioned at 60 °C for 12 h for completely removing the moisture.

The water vapor transmittance rate (WVTR) of the papers was determined by the gravimetric method [42]. A 75% RH circumstance was established inside the test chamber via the saturated sodium chloride (NaCl) aqueous solution. A circular dish (diameter of 20 mm) was filled with 15 g oven-dried calcium chloride (CaCl_2_) to maintain 0% RH. It was sealed with the paper sample and then put in the chamber, and the weight increase for the circular after 24 h was recorded. The WVTR (g/(m^2^·24 h·atm)), defined as the amount of water vapour that passes through tested material per unit area (m^2^) per unit time (24 h/day) at certain temperature and relative humidity, was calculated according to the equation below.

(2) $$\mathrm{WVTR}=\frac{\mathrm{mass}\;\mathrm{after}\;24\;h\;–\;\mathrm{initial}\;\mathrm{mass}}{\mathrm{area}\;\mathrm{of}\;\mathrm{the}\;\mathrm{testing}\;\mathrm{paper}}\;$$

A shaking flask method was used to test quantitatively the antimicrobial activities against Gram-negative bacterial *E. coli* ATCC 11229. Zero-point-two grams of paper scraps and 0.2 mL of bacterial culture (10^10^ CFU/mL) were mixed in 5 mL of normal NaCl saline (0.85 wt %) in test tubes and put into an incubator at 37 °C for 12 h. The optical density was tested by a GENESYS 10 UV–Vis spectrometer (Waltham, MA, USA) in the wavelength range of 280–600 nm. Then 0.2 mL of diluted culture (10^−4^) were seeded on the agar plate, and the plates were put into an incubator at 37 °C for 24 h. The number of colonies was counted, and three repeats were conducted for each sample. The inhibition of cell growth can be quantified by comparing with the blank sample:

Growth inhibition of cell (%) = (A − B)/A ×100(3)

where A and B are the number of colonies determined from the blank and treated samples, respectively.

Overall migration tests were performed in three food simulants: deionized water, 10% alcohol solution and 3% acetic acid, according to the EU No. 10/2011 standard [43]. Zero-point-one grams of composite films or coated paper samples were immersed in 50 mL of three food simulants and kept in a controlled chamber at 40 °C during 4 or 7 days. Subsequently, the migration levels of the ZnO NPs in the food simulants were measured using inductively-coupled plasma (ICP) spectrometry with a CETAC ASX-510 Autosampler (PerkinElmer, Waltham, MA, USA). ICP-AES analysis was conducted via a Varian Vista Pro CCD (Waltham, MA, USA) according to the literature.

### 2.4. Data Analysis

The data were analysed by Excel software (Newark, DE, USA), and the standard deviation was also calculated. All the curves were obtained by Origin 8.5 software (Wellesley, MA, USA). In order to better reflect the effect of the ZnO NP dosages on the performance of the resultant films and papers, the curves were plotted with the “B-Spline” style.

## 3. Results and Discussion

### 3.1. Miscibility of ZnO NPs in the Starch Matrix

**Figure 2:**
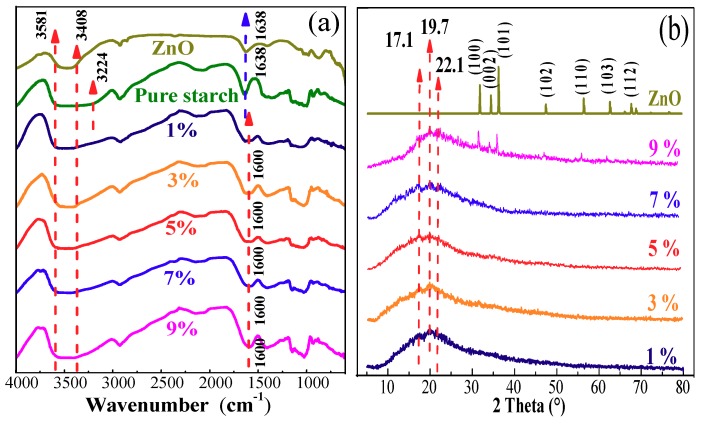
The FTIR spectra (**a**) and XRD patterns (**b**) of pure starch films and composite films with various ZnO NPs dosages.

The miscibility of ZnO NPs in the starch matrix was characterized by the FTIR and XRD measurements. In Figure 2, the characteristic peak of the –OH deformation vibration in the ZnO NPs is displayed at 1638 cm^−1^, and the absorption band ascribed to the hydrogen bonded O–H stretching vibrations appeared in the range of 3408–3581 cm^−1^ (Figure 2a) [44]. The composite films exhibited almost the same absorption band for the hydroxyl groups as the pure starch film after the addition of the ZnO NPs, demonstrating a good miscibility between the ZnO NPs and the starch, which is consistent with the reported studies [8]. The peak attributed to the vibration of hydrogen stretching had an obvious blue shift to 1600 cm^−1^ as compared with the pure starch (1638 cm^−1^), indicating the intermolecular interaction between ZnO and starch. A similar phenomenon was also observed in the reported studies, indicating the formation of certain interactions between the ZnO NPs and biopolymer matrix [45,46].

Figure 2b shows the crystallinity of the composite films and ZnO NPs. The pure ZnO particles exhibited the single-phase hexagonal wurtzite structure without any impurities [47]. The significant peaks appearing at 2θ = 31.8°, 34.5°, 36.2°, 47.8°, 56.5°, 62.8° and 68.0° correspond to the (100), (002), (101), (102), (110), (103) and (112) planes of the ZnO crystal structure, respectively. In the resultant films, the characteristic peaks at 2θ = 17.1° and 22.1° almost completely disappeared compared with the pure starch (Figure S1). This can be ascribed to the intermolecular interaction between ZnO and starch due to their excellent compatibility or the loss of the crystallinity of the starch during the gelatinization upon heating. The appearance of the phase separation can be observed clearly in the starch film owing to its aggregation as the ZnO dosage increased to 9%.

### 3.2. Hydrophobic Property and Solvent Resistance of the Films

**Figure 3:**
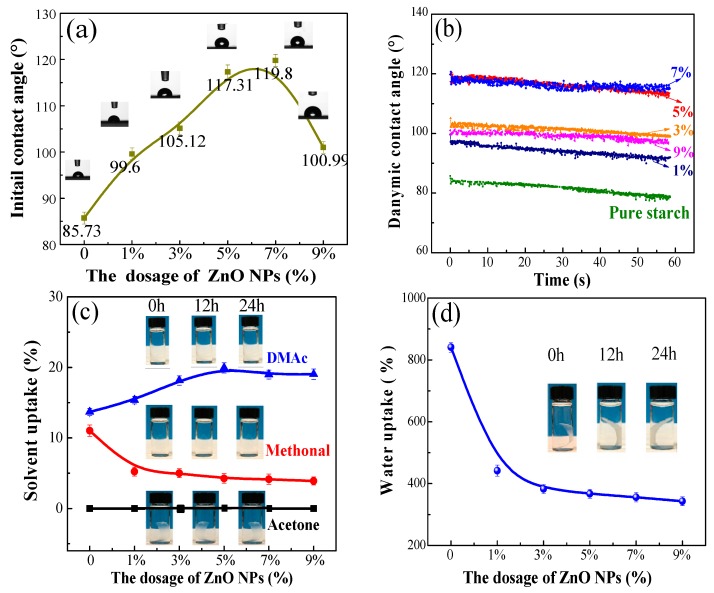
The water initial contact angles (ICA) (**a**) and dynamic contact angles (DCA) (**b**) of composite films loaded with various ZnO NP dosages. Solvent resistance of composite films with various ZnO NPs contents in different time periods against solvents of DMAc (triangle), methanol (circle), acetone (square) and H_2_O (ball). Organic solvent (**c**) and water (**d**) uptake of the composite films after 24 h of immersing. The ZnO dosage in the shown picture of the composite film is 5%. The error bar indicates the standard deviation.

Figure 3a shows the hydrophobic properties of the resultant films. As presented, increased initial or dynamic contact angles were observed on the composite films after the incorporation of the ZnO NPs as compared with the pure starch. The initial contact angle (ICA) reached a maximum value at a 7% dosage. Its hydrophobic repulsion of the ZnO can be attributed to the specific behavior of the grain boundaries according to the reports [15]. Thus, the formation of an effective electrostatic field of grain boundaries contributed to the water-repulsion phenomenon. In fact, the increased dosages of ZnO NPs contributed to the formation of the orderly micro-nano structures on the composite film, and the water droplets could not fill the grooves due to their occupation by air according to Cassie’s theory [48]. In this case, the spreading of water droplets on the composite film was effectively avoided, and the hydrophobicity was improved accordingly.

Figure 3b shows the dynamic contact angles (DCA) of the composite films. As presented, the 5% and 7% ZnO NPs dosage endowed the resultant films with the highest DCA values. This indicates that the ZnO NPs dosage was closely related to the formation of the well-organized pyramid structure within the orderly micro-nano scale. The dynamic contact angles exhibited a slight downtrend on the values in recording time. This can be ascribed to the fact that the water molecules had a readily penetration trend towards the interior of the starch films owing to their intrinsic hydrophilicity.

Several solvents (DMAc, methanol, acetone and water) were used to characterize the solvent resistance of the films. It is significant to note that no visible changes of the structure were observed after 48 h of soaking in these organic solvents (Figure 3c). The films can keep their intact shape in water despite the swelling that occurred (Figure 3d). The good solvent resistance can be ascribed to the abundant hydroxyl groups on the starch that hindered the solvent penetration in the films. The nanometer filling of the ZnO NPs also contributed to this by the formation of the compact structure.

The results of the implemented quantitative solvent uptake test are shown in Figure 3c,d. Zero uptake of acetone indicates that its absorbance only existed on the surface, and thus, acetone can be volatilized absolutely. The methanol uptake exhibited a slight downtrend with the increased ZnO dosages. This might be ascribed to the smaller size of methanol than the acetone, which can make it enter the internal parts of the films. Thus, the formation of the dense structure in the film due to the addition of the ZnO NPs decreased the amount of methanol entering the internal parts of the films and contributed to a decreased methanol uptake. As for the DMAc uptake, it varied from 15.37–19.03% as the ZnO NPs increased from 1–5%. It might be attributed to the non-volatility of DMAc and the intermolecular interaction between the DMAc and ZnO NPs. The water uptake demonstrated a sharply improved water-resistance on the resultant film: the swelling ratio for the pure starch film (Figure 3d) after 48 h of immersing was 841%, and the 5% ZnO content can decrease the water uptake to 343%.

### 3.3. Optical Properties

**Figure 4:**
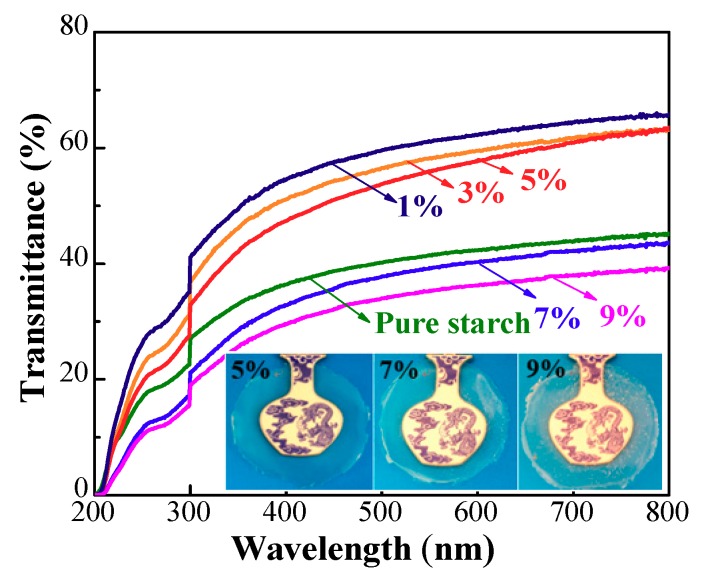
The UV-Vis spectrum of pure starch films and composite films with various dosages of ZnO NPs.

The UV–Vis measurements were used to characterize the optical properties of the films. The presented films loaded with 5%, 7% and 9% ZnO dosages demonstrated a clearly visual difference between the films (Figure 4). Visible white spots were observed on the surface at 7% content, which caused a decreased optical property, which was seen more clearly on the film with 9% ZnO NPs. Figure 4 shows the light transmittance through the films in the wavelength range of 200–800 nm. The weakened transparency of the films was clearly observed with a higher amount of ZnO NPs. As reported, the particles with diameters less than one-tenth of the visible-light wavelength did not scatter light [49]. Thus, an amount of ZnO NPs over 7% led to the sharply decreased transmittance resulting from the generated visible microscale particles (Figure 4).

### 3.4. Thermal Stabilities

**Figure 5:**
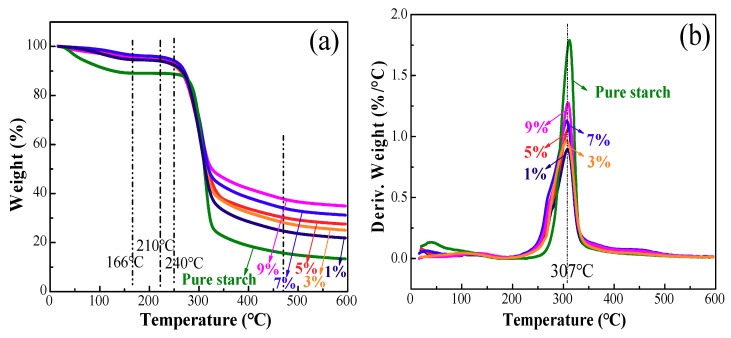
TGA (**a**) and DTG (**b**) curves for control starch film and composite films with various ZnO NP contents.

The thermal stabilities of the starch films were determined by thermogravimetric analysis (TGA), and the results are presented in Figure 5. The films exhibited two distinct stages (Figure 5a). The first weight loss of 5% in the composite films occurred below 166 °C owing to the loss of the free and combined water. Compared to the pure starch film, the composite films presented a much slower decrease in the weight at this stage. It indicates that less water was released in the composite films, possibly resulting from the enhanced hydrophobicity due to the incorporation of the ZnO NPs, which can also be observed in the derivative thermogravimetric (DTG) analysis curves (Figure 5b).

In the second stage, the weight loss in the range of 210–500 °C was attributed to the thermal degradation of the starch components, including dehydration of the sugar rings, depolymerization and decomposition of the glucose units of the polymer. As compared with that of pure starch film (240 °C), the lower initial degradation temperature (210 °C) in this stage for the composite films was ascribed to the catalytic characterization of ZnO NPs. The maximum decomposition rate of these films appeared at 307 °C (Figure 5b), and the composite film presented a lower peak height in the DTG curves compared with the pure starch films, indicating the improved thermal stability. This is also presented by the higher curve height above 307 °C and the higher “char residue”, after introducing the ZnO NPs.

### 3.5. Surface Morphology of the Films

**Figure 6:**
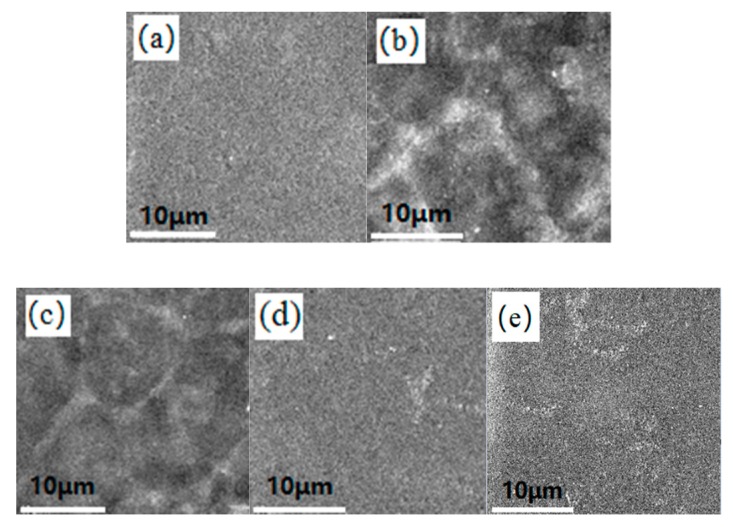
SEM micrographs of the composite films with various dosages of ZnO NPs: (**a**) 1%, (**b**) 3%, (**c**) 5%, (**d**) 7% and (**e**) 9% with 2 wt % carboxy methyl cellulose on a dry starch basis. The SEM magnification is ×2000.

The surface morphology and topography of the films were studied by SEM and AFM. As shown in the SEM micrographs (Figure S2), the heterogeneous surfaces with obvious roughness were observed at 1.5% and 2% ZnO content due to its strong aggregations in the absence of CMC. After the addition of the CMC, a homogeneous and continuous surface was seen in the composite films (Figure 6a–c), and no obvious phase separation was observed at the 1%, 3% and 5% dosages, indicating an improved miscibility of starch and ZnO. In reports, ZnO/CMC nanocomposites with good compatibility were successfully prepared [50]. This might contribute to the excellent combination between starch and ZnO NPs. The heterogeneous surface due to the aggregations of the ZnO NPs was observed at the 7% and 9% dosages (Figure 6d,e).

**Figure 7:**
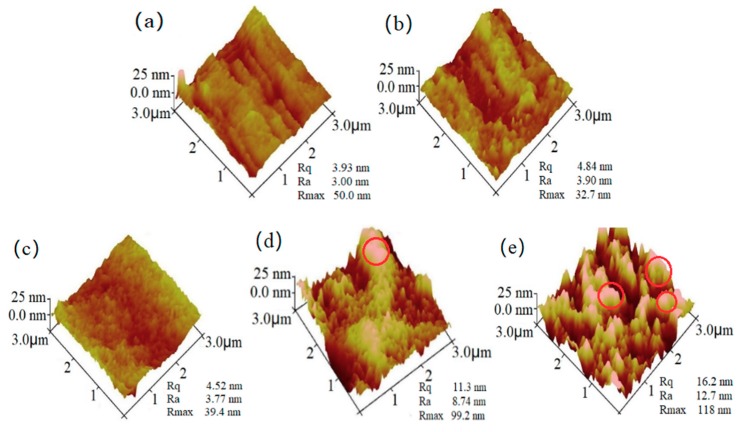
AFM micrographs of the composite films with various dosages of ZnO NPs: (**a**) 1%, (**b**) 3%, (**c**) 5%, (**d**) 7% and (**e**) 9% with 2 wt % CMC on a dry starch basis. The area of AFM images is 3 μm × 3 μm. *R*_max_ refers to the difference in height between the highest and lowest points on the surface relative to the mean plane; *R*_a_, the arithmetic average roughness of the deviations from the center plane; *R*_q_, the standard deviation of the *Z* values within the box cursor.

The 3D images from the AFM (Figure 7) clearly display the differences on the surface morphologies of these samples. The composite films revealed surfaces with grooves of increased sizes as the ZnO NPs dosage increased, demonstrating the enhanced coarseness of the film, which was also reflected by the increased *R*_a_ and *R*_q_ values in the resultant films. It is noteworthy that the *R*_max_ values at 3% and 5% ZnO content had an obvious decrease as compared with the film with 1% ZnO content, demonstrating an increased order and uniform dispersion of the ZnO NPs in the starch with a 3% and a 5% ZnO content. As the dosage further increased, a matrix with clavate units of microscale particles appeared on the starch film and thus enhanced the coarseness accordingly (Figure 7d,e).

### 3.6. Hydrophobic Property of the Coated Papers

**Figure 8:**
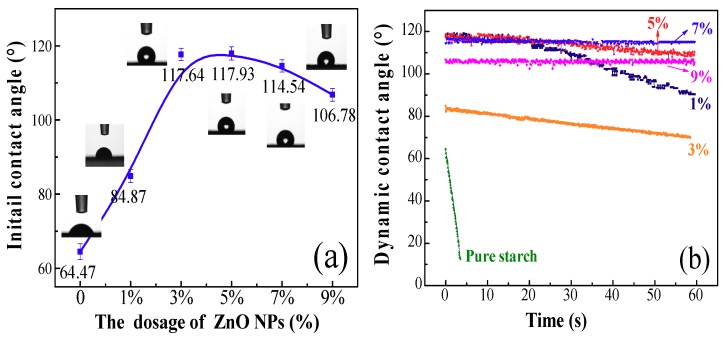
The water ICA (**a**) and DCA (**b**) of coated papers with different ZnO NP contents.

Figure 8 shows the contact angles of the coated filter papers. As seen, the initial contact angles (ICAs) in the absence of ZnO NPs were only 64.47° (Figure 8a). After the addition of the ZnO NPs, the ICAs had a significant enhancement in the range of the 3–7% dosages. The DCAs of the coated papers also exhibited excellent stabilities in the range of 5–9% ZnO content (Figure 8b). This indicates that the ZnO NPs dosage had an important influence on the formation of the micro-nano-scale structures on the papers. Figure S3 explains this phenomenon clearly: the coating fills the pores between the fibers via forming an intact and dense film, which contributes to the generated rough structure on the surface and thus hinders the spreading of water molecules.

The water vapor barrier property of the coated papers was determined to evaluate the influence of the addition of ZnO NPs (Figure S4). The pure starch paper exhibited a WVTR of 297.36 g/(m^2^·24 h·atm). By contrast, the addition of the ZnO NPs significantly decreased the WVTR from 270.73 to 234.12 g/(m^2^·24 h·atm), as the dosage varied from 1–9%. Typically, the starch coated papers had a very poor water vapor barrier property especially under high relative humidity (RH) [51]. In reports, high RH (>70%) would lead to the formation of water clusters and increase the water vapor penetration due to the increased solubility of water vapor. After the incorporation of the impervious ZnO NPs, the tortuosity in the coating layer was increased, which increased the difficulty for the water molecules to penetrate the papers. The underlying explanations are (1) the increased hydrophobicity of the papers and (2) the enhanced intermolecular force of the starch matrix.

### 3.7. Mechanical Flexible Coating with Antimicrobial Activity for Papers

The 5% ZnO content almost gave the maximum enhancement of the hydrophobicity of the resultant films and coated papers at the same time. From the results of SEM, a good miscibility between starch and ZnO NPs was observed at 5% ZnO dosage. Meanwhile, the higher ZnO led to the obvious phase separation and caused a decreased visual transmittance. Thus, 5% ZnO content was chosen in the following experiments.

**Figure 9:**
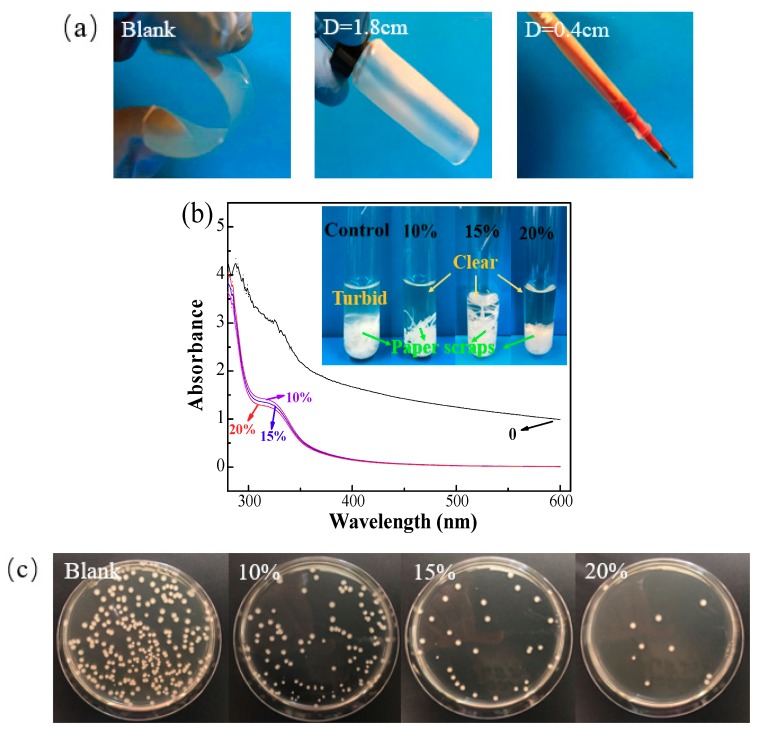
(**a**) Mechanical flexibility of the starch composite film before (blank) and after the addition of 2% CMC (D = 1.8 cm), 2% CMC and 20% antimicrobial thermal plastic starch (ATPS) (D = 0.4 cm). D represents the bending diameter of the films. (**b**) UV–Vis absorbance showing the growth levels in the supernatant of blank and coated papers with various ATPS dosages. (**c**) Pictures of the antimicrobial activities of coated papers with various ATPS dosages against *E. coli* using a shaking flask method.

Mechanical flexibility of the composite film is significant for food packaging materials [52]. After the addition of CMC, the mechanical flexibility of the composite film was sharply improved compared with the blank sample. The composite film without the addition of CMC could not be bent into a circular shape due to its rigid structure and brittleness (Figure 9a). After the addition of 2% CMC, the film exhibited good flexibility and had a bending diameter of 1.8 cm at 5% ZnO NP content. The addition of ATPS at 20% can further improve the flexibility of the composite film with a bending diameter of 0.4 cm (Figure 9b). Meanwhile, the hydrophobicity of the corresponding papers exhibited a decrease with the increased introduction of the hydrophilic ATPS, but still showed an obvious improvement in the values and stabilities of the water contact angles, as compared with the pure starch-coated papers (Figure S5).

The shaking flask method was used to characterize the antimicrobial activity of the resultant coated paper. After the addition of the guanidine-based starch (ATPS), the absorbance of *E. coli* suspension in the wavelength range of 280–600 nm had a significant decrease as compared with the blank sample, indicating its effective inhibition of the growth of *E. coli* cells. This can be clearly seen from the pictures: the solution of control (or blank) sample became turbid, while the solution of treated samples was still very clear. The results of the growth inhibition of guanidine-based starch had been studied in our previous studies [26,27,28,29,30]. Figure 9c presents the antimicrobial pictures, and the antimicrobial rates were calculated. As the ATPS dosage increased from 10% to 20%, the coated paper showed an enhanced capability to deactivate *E. coli*, and the antimicrobial rate increased from 53.5–98.1%, owing to the non-leaching characteristic of the ATPS [41]. This property for guanidine-based polymer against “leaching out” has been well demonstrated via a water rinsing experiment in our previous study [53].

### 3.8. Migration of ZnO NPs into Different Food Simulants

**Figure 10:**
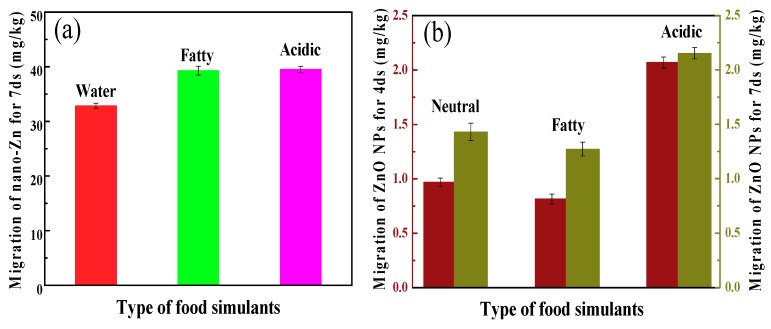
(**a**) Migration of ZnO NPs from films into different food simulants at 40 °C for four day; (**b**) migration of ZnO NPs from coated papers into different food simulants at 40 °C for four day and seven day.

Migration of ZnO NPs into the packaging material is regarded as a negative issue because excessive ZnO NPs represents a danger to human health and/or modifies the food composition. The migration tests with three kinds of food simulants (neutral, fatty and acidic) were conducted to determine the total amount of ZnO NP substances that might migrate into foodstuff from the resultant films and coated papers. The results from experiments performed in water, 10% alcohol and 3% acetic acid are displayed in Figure 10. As shown, the migration values of ZnO NPs in films (four day) were much higher than those in the coated papers, suggesting that the coated paper might be more appropriate to be used as food packaging materials.

After incubation at 40 °C, the migration levels for seven days in food simulants exhibited obvious enhancement compared with those for four days. In the case of migration in 10% alcohol, lesser migration levels were observed in comparison with water, and the maximum migration level was obtained in 3% acetic acid; however, all values were well below the overall migration legislative limits (5 mg/kg, EU No. 10/2011). This indicates that the coated papers are suited to be used as packaging materials for neutral, fatty and acidic food.

Thermodynamic properties, i.e., polarity and solubility, have a great influence on migration due to the interactions between migrants and food simulants (FS). The migrant can be retained in the polymer matrix when the migrant has a poor solubility in the FS, and the high affinity between FS and the ZnO could also lead to the adsorption by the ZnO. In addition, the sorption of organic solvents can cause the swelling of the surface of the paper matrix, which enlarges the gaps between the molecules and enhances the ZnO migration. In this work, a higher migration in acidic food simulant was observed due to the higher solubility of ZnO in comparison with water. The lesser migration in 10% alcohol can be ascribed to the decreased solubility of ZnO in this simulant. This lower migration of ZnO NPs into the food simulants can also be ascribed to the good bonding capacity and the biocompatibility of the ZnO NPs in the paper matrix.

## 4. Conclusions

In this work, a facile approach was developed to fabricate a starch-based flexible coating for food packaging papers with exceptional hydrophobicity and antimicrobial activities. Improved miscibility between ZnO NPs and starch was obtained after the addition of CMC. Excellent transmittance and uniform roughness were achieved in the resultant film at 5% ZnO NP content. Significantly, improved water ICA (117.3°) and the stability of DCAs were obtained in the resultant film and coated papers at 5% ZnO NP content. Meanwhile, the film exhibited excellent barrier capacity against methanol, acetone and water. A dramatic decrease was observed in the WVTR of the coated paper. TG (thermogravimetry) results showed the enhanced thermal properties of the resultant films, revealed by the decreased maximum decomposition rate, but the initial decomposition temperature had a slight decrease. After the addition of 20% ATPS, the composite film presented better flexibility, and the coated paper showed an excellent antimicrobial activity towards *E. coli*. Significantly, the migration of ZnO NPs was well below the overall migration legislative limit. This new type of composite coated paper could potentially be used in the food packaging fields.

## Supplementary Materials

The supplementary materials are available online at http://www.mdpi.com/2073-4360/10/11/1260/s1. Figure S1: XRD spectrum of pure starch granules; Figure S2: SEM micrographs of the starch films incorporated with (a) 1.5% and (b) 2% ZnO NPs without the addition of CMC; Figure S3: The SEM surface morphology images of the original and coating papers; Figure S4: The WVTR of coated papers with various ZnO NPs dosages; Figure S5: Water DCAs of coated papers with various ATPS dosages.
