# Supplementary material for: Starch-Based Flexible Coating for Food Packaging Paper with Exceptional Hydrophobicity and Antimicrobial Activity
Source: Polymers (Basel). 2018 Nov 13;10(11):1260. doi: 10.3390/polym10111260 (PMC6401770; doi:10.3390/polym10111260)
Supplement: Supplementary file 1 [file polymers-10-01260-s001.pdf]

Starch-based flexible coating for food packaging paper with exceptional hydrophobicity and antimicrobial activity

Shuzhen Ni [1,2], Hui Zhang [2,3], Hongqi Dai [1*], Huining Xiao[2*]

[1] Jiangsu Co-Innovation Center for Efficient Processing and Utilization of Forest Resources, Nanjing Forestry University, Nanjing, 210037, China.

[2] Department of Chemical Engineering, University of New Brunswick, Fredericton, New Brunswick E3B 5A3, Canada

[3] College of Materials Engineering, Fujian Agriculture and Forestry University, Fuzhou 350002, China

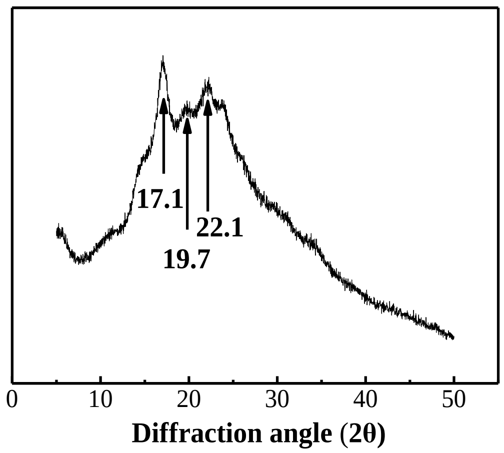

Figure S1 XRD spectrum of pure starch granules

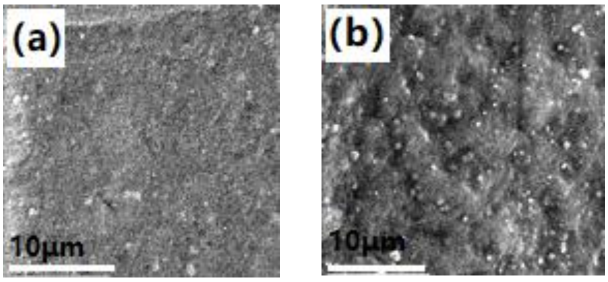

Figure S2 SEM micrographs of the starch films incorporated with (a) 1.5% and (b) 2% ZnO NPs without the addition of CMC.

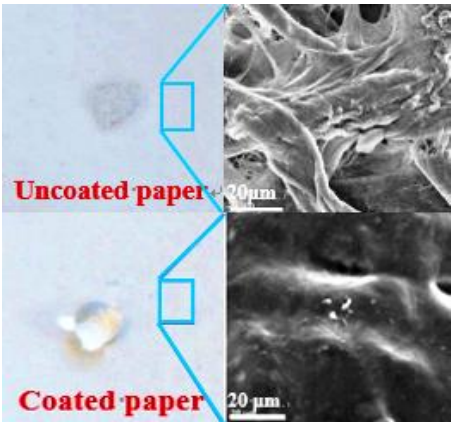

Figure S3 The SEM surface morphology images of the original and coating papers.

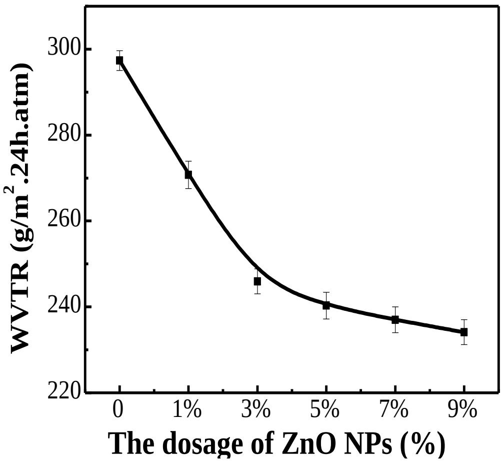

Figure S4 The WVTR of coated papers with various ZnO NPs dosages.

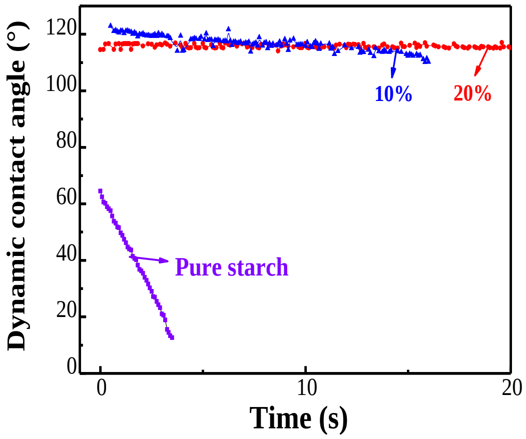

Figure. S5 Water DCAs of coated papers with various ATPS dosages.
